# Evaluation of YouTube Videos as a Source of Patient Information for Ureteric Stent Placement: A Quality Assessment Study

**DOI:** 10.3389/fsurg.2021.816222

**Published:** 2022-02-01

**Authors:** Kapil Chaudhary, Abhishek Chandna, Sudheer Kumar Devana, Aditya Prakash Sharma, Shantanu Tyagi, Shrawan K. Singh

**Affiliations:** Department of Urology, Post Graduate Institute of Medical Education and Research (PGIMER), Chandigarh, India

**Keywords:** YouTube, Double J stent (JJ stent), patient information, social media, quality assessment

## Abstract

**Objective:**

To assess the quality of YouTube videos on ureteric stent placement (USP) as a source of patient available.

**Methods:**

YouTube was searched using search terms “DJ stenting,” “Double J stenting,” and “ureteric stenting.” The initial 100 videos displayed with each of the above mentioned search terms were scrutinized. The selected videos reviewed by 3 independent consultant urologists against a pre-agreed scoring system based upon European Association of Urology (EAU) patient information sheet on ureteric stent placement. The videos were scored qualitatively and quantitatively based on the scores achieved in various domains of the scoring Performa. Data was also collected for the number of views, likes, dislikes, and time duration of each video.

**Results:**

A total of 22 videos which fulfilled the inclusion criteria were reviewed. All the videos were uploaded by healthcare organizations or healthcare websites. None of the videos were classified as “Good” based on reviewer scores and only one video was classified as “acceptable.” Fourteen videos were classified as “very poor” with a score of <5/20. General information about stents was described by majority of the studies whilst preoperative information, procedure description, danger signs, and follow up were scarcely described by most videos.

**Conclusion:**

Majority of YouTube videos on USP are of poor overall quality and lack pertinent information. This calls for creation of comprehensive and unbiased videos for patient information on USP.

## Introduction

Ever since its inception in 2005, YouTube has taken the social media platforms by storm. It attracts users all around the globe, with more than 2 billion users logged-in each month, generating billions of hours of views and videos each day (1). Its widespread availability has culminated in its utilization not only as a source of entertainment, but also a useful repository of information. YouTube has the potential to serve as a useful medium for sharing and disseminating heath related information and education (HRIE) as well. Apart from being an expansive storehouse of videos, it is also a social networking interface where the users can interact with each other. This interaction, in turn can influence decision making as well as the thought process of the users. Over 70% of internet consumers have accessed HRIE online (2–4), with a substantial proportion relying on information acquired over the internet for decision-making (3).

Urinary diversion in the form of ureteric stent placement (USP) is one of the most commonly performed urological procedures worldwide for both elective and emergency indications. Being an invasive procedure, involving the placement of a foreign body within the urinary tract, anxiety and confusion among patients is understandable. In this regard, Video-sharing platforms (VSPs) like YouTube are frequently accessed by the patients in quest of answers for their procedural doubts and to get themselves familiar with the basic technicalities of USP.

There is a plethora of content on USP available on YouTube, but unregulated uploading of videos, irrespective of medical credentials and expertise makes them vulnerable for marketing gimmicks, publicity stunts or even scientific propagandas with no level of evidence (3, 5, 6). Consequently, the viewers seeking information on USP are likely to be influenced by the content of these videos. It thus becomes imperative to ascertain the quality of such content available for general public and medical professionals alike. Through this study, we intend to assess the quality of video content available on YouTube for USP against a standard (EAU patient information sheet) (7).

## Materials and Methods

**Figure 1:**
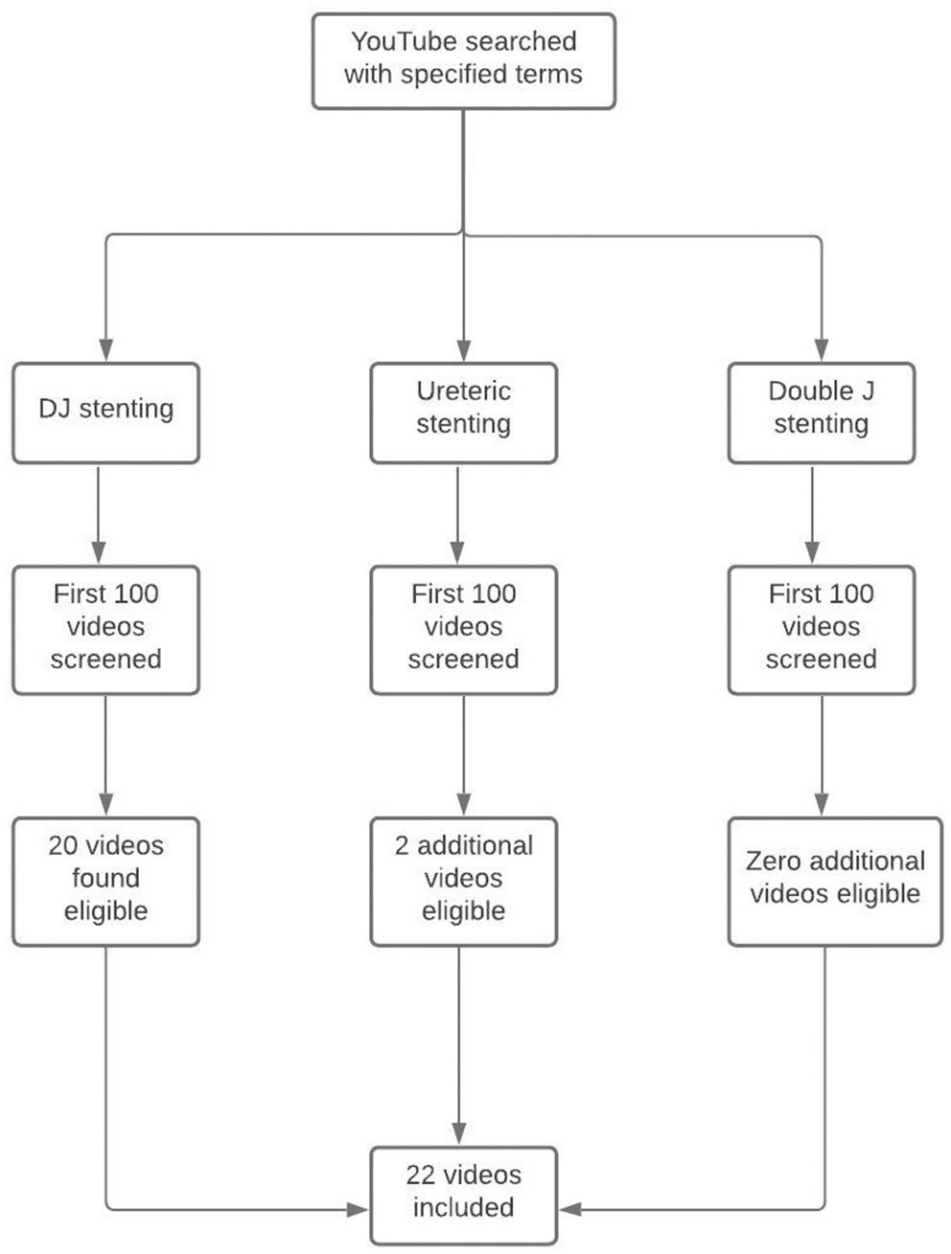
Video selection process and content.

On 21st August 2021, at Chandigarh, India, the search function on YouTube was queried with the search terms “DJ stenting,” “Double J stenting,” and “ureteric stenting.” The initial 100 videos displayed on YouTube in context of the searches made with the aforementioned terms were scrutinized. This was based on the premise that it would be highly unlikely for an individual to scroll beyond the first 100 videos. The exclusion criteria were videos with patient testimonials, videos directed toward the surgeon or urologist for placement of ureteric stent, videos in languages other than English, videos not focused on DJ stenting and videos without verbal audio. All the enlisted videos were scrutinized by a consultant urologist (KC) and 22 videos were found suitable to be included in the study (Figure 1). No filters were applied for duration or for date of uploading. Data was collected for total number of views, video duration, number of likes, dislikes, number of comments, and source of the video.

The selected videos were thereafter reviewed independently by three urology consultants (KC, SK, and AS) using a pre-agreed scoring system as listed in Table 1. These criteria were based upon the European Association of Urology (EAU) patient information sheet on double-J stent placement (7). This was deemed as a good and reliable source of patient information, that an ideal video should also contain. The criteria had a total score of 20 and a qualitative rating was awarded based on the reviewer's score: “Very poor” (0–5), “Poor” (6–10), “Acceptable” (11–15), and “Good” (16–20). The reviewer's ratings were then compared.

**Table 1:** Scoring criteria for videos, based on European Association of Urology patient information sheet (1 point for each criteria).

**Serial No**.	**Criteria**	**Information**	**Maximum Score**
1.	Information on DJS	Description of DJS Indication of DJS	2
2.	Preoperative information	Need for urine culture Withholding anti-platelets/anti-coagulants Anesthesia-local vs. general	3
3	Procedure description	Positioning Use of cystoscope Use of contrast Radiation exposure Stent placement	5
4.	Stent related symptoms	Burning micturition Blood tinged urine Lower abdominal discomfort Frequent micturition	4
5.	Danger signs	High grade fever Urine retention Gross haematuria Severe pain despite analgesics	4
6.	Follow-up	Timing of stent removal How is stent removed	2
	Total score	20	

Data was stored in SPSS program (Version 23, IBM corporation; Armonk, NY, USA). The data was expressed as numbers and percentages. To assess the reliability of inter-observer assessment, intra-class correlation coefficient was calculated and qualitative ratings were evaluated by utilizing Fleiss kappa. All statistical tests were two-sided and were performed at a significance level of *p* < 0.05.

## Results

A total of 22 videos were found to fulfill the inclusion criteria. The mean time duration of the videos was 158.6 ± 128.1 s (20–464 s). The total number of views was 14,47,832 with a median of 34,500 views. The number of likes received by a video ranged from zero to 971 and dislikes ranged from 0 to 93 (Table 2). All the videos were uploaded by healthcare organizations or healthcare websites. The median number of comments per video were 7 (0–48.25).

**Table 2:** YouTube characteristics of the analyzed videos.

**Variables**	**Values**
Number of videos	22
Length of the video (mean in seconds)	158.6 ± 128.1
Likes per video (median)^*^	109 (6–293.5)
Dislikes per video (median)^*^	10 (1.75–37.75)
Comments (median)^*^	7 (0–48.25)
Views (median)^*^	34,500 (7,900–95,100)
**Video ratings based on average scores by reviewers**	
Good (16–20)	0
Acceptable (11–15)	1
Poor (6–10)	7
Very poor (0–5)	14

* *Median values are reported with interquartile range in brackets*.

The kappa statistic for inter-observer correlation was 0.698, and intra-class correlation was 0.925. Of the 22 videos, none of the videos received a rating of “Good” and 14 of the videos received a rating of “Very poor” based on the reviewer's scores (Table 3). The mean average scores for all the reviewers was <5 (4.59, 4.45, and 4.55 for I, II, and III consultant, respectively) falling in the “very poor” category (Table 2). Only one video was unanimously rated as more than 10 by all reviewers, culminating to a qualitative category of “acceptable,” while none of the videos was awarded a score of >15 by any of the reviewers. General information about the stent like its description and indication for placement was described by most of the studies (14/22; Supplementary Table 1). Information on preoperative work-up, stent related symptoms, procedural description, and follow-up was not described by majority of the videos (Supplementary Table 1).

**Table 3:** Mean scores of each consultant in different scoring criteria.

**Scoring criteria (maximum score)**	**Consultant I**	**Consultant II**	**Consultant III**
Information on DJ stent (2)	1.59	1.55	1.77
Preoperative information (3)	0.23	0.14	0.18
Procedural description (5)	0.73	0.5	0.55
Stent related symptoms (4)	1	1	1.05
Danger signs (4)	0.32	0.5	0.32
Follow-up (2)	0.73	0.77	0.68
Total score (20)	4.59	4.45	4.55

## Discussion

The present study analyzed the quality of health related information on ureteric stent placement available to patients via YouTube videos. Majority of the videos failed to provide good quality information about USP to the patients, ranking as “very poor” when rated by 3 independent consultant urologists. Though a good number of videos provided basic information about ureteric stents, most of them failed at explaining about the procedure, preoperative requirements, complications, danger signs as well as the follow-up for such stents. None of the videos confirmed to all the attributes outlined by the EAU patient information sheet for ureteric stenting.

Only one video received a total score of more than 10 by all the reviewers, lasting for 7 min 44 s, yet it failed to describe the procedure and the preoperative concerns. This video was well above the mean video length of 158.6 ± 128.1 s. The two videos with the highest number of views were rated as “poor” by all the 3 reviewers. Fourteen videos received a “very poor” rating by all reviewers and had a mean score of <5 thus questioning the true utility of YouTube videos as a reliable source of patient education and information.

The current medical practice is undergoing a change toward shared decision making for which patient's information and education constitutes a vital cornerstone. Social media outlets like YouTube, Twitter, and Facebook play an important role in dissemination and distribution of information, considering their huge number of subscribers and views per day. With widespread access to internet services, the patient and their attendants seek information regarding their symptoms, procedures, healthcare facilities, and the credentials of healthcare provider prior to scheduled appointment.

Recently, a myriad of studies pertaining to urological diseases and procedures have analyzed the reliability of YouTube videos as a source of medical information for patients (8–15). These studies, spanning across benign prostatic hyperplasia, stone treatment (15, 16), infertility (17), and erectile dysfunction (9) observed that YouTube videos had low quality of content, provided unreliable or false information and were subject to commercial bias. To best of our knowledge of English literature, ours is the only study examining the role of such videos for ureteric stent placement and echoes the concerns raised by the existing studies. Only one study examining the quality and reliability of YouTube videos on pelvic floor muscle training observed most of the studies to be useful, albeit moderate in reliability, quality, and accuracy (18). Loeb (11) examined the existing literature for impact of social media on information about various benign and malignant urological conditions. They concluded that majority of the available information was biased, inadequate, misinformative, and commercially sponsored.

Comments, likes, and dislikes have also been found to have an important impact on the viewership, in turn influencing the viewers, irrespective of who comments on the videos and the authority/knowledge of the person. The median number of comments per video in the present study were 7 (range 0–219). A notable example of comments influencing public opinion is the human papillomavirus vaccination promotion on YouTube, where glaringly inaccurate information and propaganda was circulated in the form of negative commentary (19). Moreover, negative videos were liked more than positive ones by the viewers (20).

Popularity often measured in terms of view counts and/or public ratings is also an important concept in assessing the quality of YouTube videos. Unlike the focus on the assessment of the quality of content, which relies on human judgement and evaluation, view count, or video views per day are quantitative measures that are readily accessible for each video on YouTube. However, some videos have higher view counts due to marketing campaigns, viral effects, duration of availability of the video, or the video being linked from several webpages. Users need to be aware that frequency of views may be manipulated by parties with specific agendas to achieve its “perceived” popularity. Similarly, in the present study, 5 videos had more than 100,000 views each; all of them were qualified as “poor” source of information unanimously by the reviewers. The only study with a score >10, garnered 76,000 views, despite being around for over 2 years. This was probably due to its long duration (7 min 44 s) when compared to the rest of the videos.

The centers for disease control and prevention (CDC) has specified guidelines for publishing videos on its YouTube channel (21). However, these apply only to CDCs YouTube channel and are not uniformly followed. The Health on Net (HON) foundation also has attempted to standardize the information available online so that reliable, comprehensive, and trustworthy information is available to the public at large. The HON code of conduct (HONcode), which serves as a guarantee for reliable information, is accredited to websites ensuring basic ethical standards and where the source and purpose of data being presented in available and reliable (22). Unfortunately, no such system of accreditation exists for YouTube videos at present. This highlights the unexplored opportunities for medical professionals to produce high-quality, patient centered comprehensive informational videos online.

The present study has a few limitations. The results were limited to the first 100 videos only, which may have excluded some videos, but it is unlikely that viewers may go beyond first 100 videos of search results. Secondly, videos available only on YouTube were analyzed, and those on other VSP platforms were not included owing to the study design. YouTube, being a widely and openly available source of information was chosen as a source for the present study. Another drawback of the study is its restriction to English language videos only, leading to exclusion of a few high-quality videos.

YouTube provides an unparalleled resource of free and open access videos which may provide information to patients, aiding in education and decision making. However, they are often unregulated, biased and may contain insufficient information for patient education when compared to professional information such as EAU patient information platform. In summary, videos describing USP on YouTube should not be recommended for patient education as they fail to address a glaring majority of important aspects about ureteric stents. The results of this study underline the importance of thorough communication between the medical professional and the patient as well as calls out for regulation on the content available on such VSPs in order to provide comprehensive information to the patients. Promotion of well-balanced, unbiased and evidence-based online information platforms is the need of the hour.

## Conclusion

Majority of the videos on ureteric stent placement available on YouTube are of poor overall quality and lack important information. This presents a risk of exposure to misinformation and calls for creation of unbiased and comprehensive videos for patient information on ureteric stent placement.

## Data Availability Statement

The original contributions presented in the study are included in the article/Supplementary Material, further inquiries can be directed to the corresponding author/s.

## Author Contributions

KC: protocol development, data collection, data analysis, manuscript writing, and editing. AC: data collection, data analysis, manuscript writing, and editing. SK and AS: protocol development, manuscript writing, and editing. ST: protocol development and data collection. SS: data analysis, manuscript writing, and editing. All authors contributed to the article and approved the submitted version.

## Conflict of Interest

The authors declare that the research was conducted in the absence of any commercial or financial relationships that could be construed as a potential conflict of interest.

## Publisher's Note

All claims expressed in this article are solely those of the authors and do not necessarily represent those of their affiliated organizations, or those of the publisher, the editors and the reviewers. Any product that may be evaluated in this article, or claim that may be made by its manufacturer, is not guaranteed or endorsed by the publisher.
