# Supplementary material for: Evaluation of YouTube Videos as a Source of Patient Information for Ureteric Stent Placement: A Quality Assessment Study
Source: Front Surg. 2022 Feb 1;8:816222. doi: 10.3389/fsurg.2021.816222 (PMC8843829; doi:10.3389/fsurg.2021.816222)
Supplement: Supplementary file 1 [file Table_1.DOCX]

| Criteria  (Score given/maximum score) | Consultant I | Consultant II | Consultant III |
| --- | --- | --- | --- |
| Information on DJS   - 0/2 - 1/2 - 2/2 | 1  7  14 | 2  6  14 | 0  5  17 |
| Preoperative Information   - 0/3 - 1/3 - 2/3 - 3/3 | 17 5  0  0 | 19  3  0  0 | 18  4  0  0 |
| Procedure Description   - 0/5 - 1/5 - 2/5 - 3/5 - 4/5 - 5/5 | 16  1  2  1  2  0 | 18  0  2  1  1  0 | 16  2  2  2  0  0 |
| Stent related symptoms   - 0/4 - 1/4 - 2/4 - 3/4 - 4/4 | 15  0  3  0  4 | 14  1  2  3  2 | 15  0  1  3  3 |
| Danger Signs   - 0/4 - 1/4 - 2/4 - 3/4 - 4/4 | 19  1  1  0  1 | 17  2  1  1  1 | 20  0  0  1  1 |
| Follow-up   - 0/2 - 1/2 - 2/2 | 9  10  3 | 9  9  4 | 11  7  4 |

Supplementary table 1: Distribution of the number of videos as per the scores attained across the various categories

Supplementary table 2 : Details of videos reviewed for the study

| S.No | Title | Source | Time | likes | dislikes | views | comments |
| --- | --- | --- | --- | --- | --- | --- | --- |
| 1 | What is a ureteral stent ? | Pacific norwest urology specialists | 2:54 | 620 | 54 | 170k | 219 |
| 2 | Ureteric stent misconceptions | Pacific norwest urology specialists | 4:50 | 55 | 4 | 1.99k | 6 |
| 3 | JJ stent (removal of kidney stones) | EAU | 2:25 | 971 | 93 | 293K | 40 |
| 4 | Ureteral stent placement | PreOp.com Patient Engagement-Patient Education | 7:44 | 291 | 36 | 76k | 42 |
| 5 | Why do stents cause some kidney stone patients pain? | UW Medicine | 2:17 | 142 | 7 | 29k | 0 |
| 6 | Treatment: Stent pain that can occur after kidney stone surgery | Cleveland clinic | 0:52 | 152 | 27 | 64k | 102 |
| 7 | What is a ureteral stent ? | Boston scientific urology | 0:20 | 0 | 0 | 24k | 0 |
| 8 | What are the side effects of a ureteral stent ? | Stonesprings hospital center | 1:41 | 41 | 5 | 10k | 49 |
| 9 | Stents can be a nuisance | Center4menswomensuro | 3:04 | 0 | 0 | 951 | 0 |
| 10 | Why does it hurt when I urinate with a ureteral stent? | Stone springs hospital center | 1:52 | 88 | 8 | 23k | 48 |
| 11 | Ureteroscopy (URS) | Boston scientific urology | 1:26 | 0 | 0 | 92.8k | 0 |
| 12 | Kidney stent removal – did it hurt (see inside my bladder) | Transplant fitness | 7:09 | 236 | 32 | 47k | 116 |
| 13 | Endoscopic DJ stents video EN | BIOTEQUE2010 | 3:56 | 46 | 12 | 24K | 1 |
| 14 | What is a ureteral stent ? how is a stent placed and removed? Dr Manohar T | Doctors’ circle – worlds largest health platform | 1:40 | 301 | 77 | 158k | 29 |
| 15 | What is a ureteral stent How is a stent placed and removed? Dr Santosh Bethur | Doctors’ circle – worlds largest health platform | 1:56 | 130 | 43 | 72k | 8 |
| 16 | Removal of kidney stones: URS | European association of urology | 2:53 | 742 | 51 | 206k | 15 |
| 17 | Kidney stones surgery- PreOp patient education and patient engagement | PreOp.com Patient Engagement-Patient Education | 6:10 | 368 | 30 | 102k | 76 |
| 18 | Removal of kidney stones: PCNL | European association of urology | 2:44 | 183 | 17 | 40k | 3 |
| 19 | What are some side effects of a ureteral stent | Los Robles Regional Medical Center | 0:29 | 8 | 2 | 3.7k | 1 |
| 20 | Why is ureteric stent inserted? | kidneystoneclinic | 0:30 | 7 | 3 | 9.3k | 0 |
| 21 | What is a ureteral stent? – Frankfort Regional Medical Center | Frankfort Regional Medical Center | 0:40 | 2 | 0 | 239 | 0 |
| 22 | What is a ureteral stent? | SA Health | 0:38 | 3 | 1 | 852 | 0 |
